# Diabetes Self-Care Activities and Their Relationship With Glycemic Control in Patients Visiting Hayatabad Medical Complex, Peshawar

**DOI:** 10.7759/cureus.42741

**Published:** 2023-07-31

**Authors:** Wagmah Javed Khan, Muhammad Arsalan, Wardah Javed Khan

**Affiliations:** 1 Internal Medicine, Hayatabad Medical Complex Peshawar, Peshawar, PAK

**Keywords:** glycemic control, glycated hemoglobin (hba1c), medication adherence, type 2 diabetes, diabetes self-care activities

## Abstract

Background

A significant portion of the Pakistani population is affected by diabetes, which has emerged as a global healthcare concern.

Objective

This study aimed to assess the correlation between glycemic control in diabetes patients and their engagement in diabetes self-care activities (DSCA).

Methodology

Cross-sectional research was conducted at Hayatabad Medical Complex in Peshawar between June 2019 and May 2020. A total of 280 carefully selected patients with type 2 diabetes mellitus (T2DM) were included. Data collection involved an interviewer-administered questionnaire encompassing sociodemographic information, diabetes-related data, and the summary of the Diabetes Self-Care Activities (SDSCA) scale. Descriptive statistics and Pearson's chi-square test were employed for data analysis.

Results

The study observed that the majority of participants (40.36%) were females, and the age range of the participants was between 42 and 53 years (68.22%). According to the study, 55.00% of participants had a normal body mass index (BMI), and 71.08% of participants had diabetes in their family. Regarding glycemic control, 55.71% of individuals exhibited good control based on fasting blood sugar (FBS) levels while 74.64% showed poor control according to hemoglobin A1C (HbA1c) values. HbA1c was substantially linked with a general diet (healthy eating plan), physical activities, and adherence to medication ((odds ratios (OR): 3.12), (95% confidence interval (CI): 1.02 - 8.78), (P value: 0.031)); ((OR: 2.19, 95%), (CI:1.18 - 3.79), (P value: 0.003)); ((OR: 2.85), (95% CI: 1.22 - 6.59), P value: 0.021)).

Conclusion

The findings indicated that health professionals need to create health education programs on DSCA in order to increase DSCA adherence in people with T2DM while maintaining glycemic control.

## Introduction

In Pakistan, diabetes affects a considerable portion of the population, with 26.7% of adults being affected by the condition in 2022, as reported by the International Diabetes Federation. This high prevalence creates a significant healthcare burden for the country. Achieving optimal glycemic control is necessary for good diabetes management and a consequent reduction in complications. Therefore, it becomes crucial to comprehend how diabetes self-care activities (DSCA) and glycemic control are related. DSCA covers a range of actions, including taking medications as prescribed, checking blood sugar levels, choosing healthy foods, getting regular exercise, managing stress, and obtaining the right medical assistance. However, Pakistan's distinct cultural, societal, and economic characteristics might affect how well these activities work and how they affect glycemic control [1-4]. The successful implementation of self-care actions may be hampered by elements including ingrained food habits, restricted access to healthcare services, and cultural views toward diabetes. Therefore, researching these obstacles and adjusting therapies as necessary might help the Pakistani population's management of diabetes and glycemic control [5-7].

The goal of this study was to determine the relationship between DSCA and glycemic control, revealing crucial information on the factors influencing self-care behaviors and their implications for the management of diabetes. In the end, our research aims to empower people, enabling them to take charge of their health and perhaps lowering the risk of complications from diabetes. This research can aid in the creation of focused treatments and personalized strategies by finding effective self-care practices unique to the Pakistani setting, thereby improving the quality of life for people with type 2 diabetes mellitus (T2DM).

## Materials and methods

Study setting and duration

This cross-sectional study was conducted at Hayatabad Medical Complex Peshawar, Pakistan, from June 2019 and May 2020, and 280 adults with T2DM were selected. Adults with T2DM who were 18 years of age or older and had the diagnosis for at least six months were sought out for this study. The inclusion criteria gave people enough time to become used to their condition and take part in self-care for diabetes. To guarantee a more homogenous sample and reliable results, however, adults who were extremely ill, with a recent diabetes diagnosis of a minimum of six months, were excluded from the study along with individuals who had physical or mental disabilities, pregnancy, and recent diabetes diagnosis.

Ethical statement

The Ethics Review Committee of the Hayatabad Medical Complex Hospital in Peshawar, Pakistan, provided the research approval under endorsement number 27220 in May 2019.

Data collection

The researcher's interviewer-administered questionnaire for this study was based on the Summary of Diabetes Self-Care Activities (SDSCA) measure, which is often used to evaluate diabetes patients' behaviors about self-care [8]. The survey was divided into three sections. The first section collected sociodemographic data, the second section collected diabetes-related data (including the duration of the condition, a person's family history, the results of hemoglobin A1C (HbA1c) tests, etc.), and the third section contained the validated SDSCA scale. The SDSCA scale evaluated self-care practices such as food, exercise, blood glucose monitoring, medication adherence, and foot care. Participants answered questions on how frequently they had engaged in these activities during the previous seven days, taking into account, if necessary, the days before any sickness had developed. The seven-day cut-off was chosen to standardize data collection, capture recent behavior, and minimize recall biases while considering any impact of illness on participants' activities. Ten people with T2DM validated the survey that was provided before the trial to make sure it was acceptable, feasible, understandable, and suitable; these individuals weren't included in the actual study.

Data analysis

Sample characteristics were evaluated using descriptive statistics and SPSS (Statistical Package for Social Sciences) 20.0 version (IBM Corp., Armonk, NY). Pearson's chi-square test was used to examine the relationships between the degree of self-care practices, HbA1c, fasting blood sugar (FBS), body mass index (BMI), and socio-demographic characteristics. For all tests, a confidence interval of 95% and a probability of 0.05 were regarded as statistically significant.

## Results

Table 1 provides an overview of key socioeconomic and demographic features common to individuals with T2DM. According to the gender distribution, 31.78% of people were male and 68.22% of people were female. The bulk of participants were between the ages of 42 and 53 (40.36%), followed by the 54 to 65 age range (32.50%). According to the participant's marital status, 83.21% of them were married, 6.79% were single, and 10.00% were in another category. The biggest percentage (42.50%) of people had at least an intermediate education, followed by graduates (33.21%), in terms of educational standing. In terms of occupation, housewives (45.36%) made up the biggest category, followed by retirees (24.64%). A monthly income of between 40,001 and 60,000 Pakistani rupees was earned by 53.21% of the families.

**Table 1:** Socio-demographic profile of patients with T2DM (n=280) T2DM: type 2 diabetes mellitus

Variables	Frequency	Percent (%)
Gender		
Male	89	31.78
Female	191	68.22
Age in years		
18-29	6	2.14
30-41	49	17.50
42-53	113	40.36
54-65	91	32.50
Above 65	21	7.50
Marital status		
Single	19	6.79
Married	233	83.21
Other	28	10.00
Educational status		
Not attained to the school	4	1.43
Primary	13	4.64
High school	51	18.21
Intermediate (HSSC)	119	42.50
Graduate	93	33.21
Occupation		
Unemployed	8	2.86
Retired	69	24.64
Housewife	127	45.36
Professional	31	11.07
Vendors & sellers	18	6.43
Technical & clerical	13	4.64
Skilled manual workers	10	3.57
Unskilled manual workers	4	1.43
Average monthly income for the family		
Equal to 40,000 PKR (Pakistani Rupees)	29	10.36
40,001 PKR – 60,000 PKR	149	53.21
60,001 PKR – 80, 000 PKR	85	30.36
More than 80,000 PKR	17	6.07

Important characteristics and health-related data of those with T2DM are shown in Table 2. The majority (71.08%) were found to have a family history of T2DM, suggesting that there may be a genetic component. The duration of diabetes varied, with a considerable majority (46.43%) having it for two to 10 years. According to BMI distribution, a sizable portion (55.00%) of people were considered normal, 30.71% were considered underweight, and 0.72% were considered obese. Data showed that more than half (55.71%) had good blood sugar control, whereas the remaining (44.29%) showed poor control, which was an important factor. Similar results were shown for HbA1c readings, which showed that a sizable majority (74.64%) had poor control and a smaller portion (25.36%) had good control. When it came to medication, the majority (75.71%) solely used oral tablets.

**Table 2:** Comprehensive diabetes information for T2DM patients BMI: body mass index; HbA1c: hemoglobin A1C; FBS: fasting blood sugar; T2DM: type 2 diabetes mellitus

Variables	Number of Patients (n)	Percentage (%)
Family-related background for T2DM		
Yes	199	71.08
No	78	27.85
Don’t know	3	1.07
Diabetic condition duration		
Six - Twelve months	7	2.50
One - Two years	26	9.29
Two - Ten years	130	46.43
More than 10 years	117	41.78
BMI		
Underweight (less than 18.50)	86	30.71
Healthy weight	154	55.00
Overweight	38	13.57
Obesity (greater than and equal to 30)	2	0.72
Medicine		
Insulin	9	3.22
Oral and insulin	59	21.07
Oral pills	212	75.71
HbA1c level		
Ineffective control (≥ 7%)	209	74.64
Optimal control (< 7%)	71	25.36
FBS value		
Ineffective control (> 126 mg/dl)	124	44.29
Optimal control (≤ 126 mg/dl)	156	55.71

The link between DSCA and glycemic control in people with T2DM is summarized in Table 3. FBS and HbA1c levels, as well as odds ratios (OR) and 95% confidence intervals (CI) for the relationships, are some of the factors that are included. Participants who followed the general and particular diets had better glycemic control than those who did not practice good self-care in these areas. HbA1c and overall food consumption were significantly linked ((OR: 3.12), (95% CI: 1.02 - 8.78), P value: 0.031)). Physical activity was also significantly linked with HbA1c ((OR: 2.19, 95%), (CI:1.18 - 3.79), (P value: 0.003)); FBS was strongly correlated with both the overall diet and the specific diet ((OR: 3.46), (95% CI: 1.69 - 7.97), (P value: 0.001)) and ((OR: 2.36), (95% CI: 1.46 - 3.77), (P value: 0.001)), respectively). There was a significant correlation between adherence to medication and both HbA1c and FBS ((OR: 2.85), (95% CI: 1.22 - 6.59), P value: 0.021)) and ((OR: 1.88), (95% CI: 1.05 - 3.29), (P value: 0.021)), respectively). Glycemic control and other DSCA outcomes are not significantly associated.

**Table 3:** Relationship between DSCA and glycemic control SMBG: self-monitoring of blood glucose; DSCA: Diabetes Self-Care Activities * p < 0.05, ** p < 0.001 level of significance

Variables	Glycemic control (HbA1c)					Glycemic control (FBS)				
	Optimal control	Ineffective control	OR	CI: 95%		Optimal control	Ineffective control	OR	95% CI	
				Minor	Higher				Lower	Upper
Physical exercise										
Optimal (3 to 7 days)	46*	110	2.19	1.18	3.79	85	71	1.51	0.37	1.71
Ineffective (0 to 2 days)	17	107				63	61			
Diet (General)										
Optimal (3 to 7 days)	60*	188	3.12	1.02	8.78	143**	109	3.46	1.69	7.97
Ineffective (0 to 2 days)	3	29				5	23			
Diet (Specific)										
Optimal (3 to 7 days)	31	89	1.37	0.80	2.43	79**	41	2.36	1.46	3.77
Ineffective (0 to 2 days)	32	128				69	91			
Foot treatment										
Optimal (3 to 7 days)	31	90	1.32	0.78	2.28	67	54	1.16	0.71	1.81
Ineffective (0 to 2 days)	32	127				81	78			
SMBG										
Optimal (3 to 7 days)	5	44	0.55	0.23	1.17	27	22	1.03	0.58	1.89
Ineffective (0 to 2 days)	58	173				121	110			
Medication adherence										
Optimal (complete week)	60**	168	2.85	1.22	6.59	129**	101	1.88	1.05	3.29
Ineffective (0 to 6 Days)	4	48				19	31			

## Discussion

The research study conducted at Hayatabad Medical Complex in Peshawar, Pakistan, aimed to investigate the relationship between DSCA and glycemic control among patients with T2DM. The findings from this study provide valuable insights into the factors influencing self-care practices and their impact on diabetes management within the Pakistani population. The results of this study clearly show that while the majority of patients had low HbA1c values, more than half of the subjects had excellent FBS levels. Adults with T2DM have been shown to have poor glycemic control, according to the findings of other investigations [9-11].

In this study, 71.08% of the participants had a family history of diabetes. Another cross-sectional nationwide study of 4890 people in Sri Lanka revealed that patients with a family history of diabetes had a considerably greater prevalence of the disease (29.0%) than patients without a family history (7.2%) [12]. Additionally, the results of the current study showed that over half, i.e., 55.0% had a standard BMI. This result is in line with the results of another research, which showed that 47.8% of the subjects had a standard BMI [13].

Even though the majority of participants in the current research (88.57%) followed a general diet for three or more days in the previous seven (healthy eating plan), 57.14% did not follow a specific diet for three or more days in the previous seven. Because the Pakistani diet is high in calories from carbohydrates, the main message for dietary change should be on reducing rice intake while increasing quantities of vegetables and green leaves [14]. However, another study found that 54.2% of participants had ineffective eating habits linked to type 2 diabetes and that the majority of people (69.2%) used some food constraint [15].

The present research also showed that 55.71% of participants adhered to an exercise schedule on three or more days during the course of the previous seven days. But according to different research, the majority of individuals (67.18%) did not engage in any type of physical activity [16]. In this study, over 50% of individuals were not involved in self-monitoring of blood glucose (SMBG) for a single day. Another research found that only seven (4.26%) of the 164 persons with T2DM had engaged in SMBG at least monthly [17].

In the previous seven days, over half (56.78%) did not take care of their feet on even a single day. Only 43.22% of people in the preceding week adhered to foot treatment for three or above days. Similar to the current study, a different investigation revealed that a significant majority of patients (71.2%) did not take good care of their feet [15]. The current research displayed that most participants (81.42%) regularly took their medications; a different study's findings revealed that among the participants, 69.37% displayed poor medication practices [15], while the study conducted by Omani found that adherence to medication was 78%, with three or more days in the previous seven days [18].

Study limitations

Although the study offers insightful information, it is vital to recognize its limits. First, because the sample was restricted to patients at the Hayatabad Medical Complex in Peshawar, Pakistan, it may be difficult to extrapolate the results to a larger population. The study's external validity would be improved by including individuals from various medical facilities. Second, using only self-reported data exposes you to memory and social desirability biases, among other possible biases. Data may be more accurate if objective metrics are used such as wearable technology or medical records. Future studies that address these issues will improve our knowledge of the connection between DSCA and glycemic management in T2DM patients.

## Conclusions

The results showed that most patients adhered to the recommended diet; however, more than half failed to follow a particular diabetes diet. Many folks didn't stick to their fitness routine for even one day. Over 50% of the individuals were not involved in SMBG for a single day. In the last seven days, over half of the patients didn't even attempt to take care of their feet once. The majority of participants said they routinely took the prescribed prescription. While the majority of subjects had low HbA1c values, nearly half of them had excellent fasting blood glucose levels. Diet, both general and particular, was significantly linked to FBS. HbA1C and physical activity were highly correlated. Compared to male participants, female individuals showed greater adherence to foot care procedures. Age and physical activity were significantly correlated. Thus, all of these findings suggest that persons who had good HbA1c and FBS management reported greater DSCA adherence.
